# Pattern and outcome of acute poisoning cases in a tertiary care hospital in Karnataka, India

**DOI:** 10.4103/0972-5229.58541

**Published:** 2009

**Authors:** K. N. Ramesha, Krishnamurthy B. H. Rao, Ganesh S. Kumar

**Affiliations:** **From:** Department of Neurology, Sree Chitra Tirunal Institute for Medical Sciences and Technology Trivandrum, Kerala, India; 1 Department of General Medicine, Kasturba Medical College, Mangalore, Karnataka, India; 2 Department of Community Medicine, Kasturba Medical College, Mangalore, Karnataka, India

**Keywords:** Acute poisoning, pattern and outcome, tertiary care hospital

## Abstract

**Background and Objective:**

Acute poisoning is a medical emergency. It is important to know the nature, severity and outcome of acute poisoning cases in order to take up appropriate planning, prevention and management techniques. This study aimed to assess the pattern and outcome of acute poisoning cases in a tertiary care hospital in Karnataka.

**Materials and Methods:**

This is a retrospective hospital record-based study conducted in a tertiary care hospital attached to a medical institution in Karnataka. The study included 136 cases and data regarding age, sex, time elapsed after intake; circumstances of poisoning, name of the poisonous substance, chemical type, duration of hospitalization, severity and outcome were collected in the prestructured proforma.

**Results:**

Incidence was more common among males (75.4%) compared to females (24.3). Most cases of acute poisoning presented among 20- to 29-year age group (31.2%) followed by 12- to 19-year age group (30.2%). A majority of poisoning cases (36.0%) were due to organophosphorus compound (OPC). Total mortality was found to be 15.4%. Mortality rate due to corrosives was significantly high compared with OPC poisoning (χ^2^ = 4.12, *P* = 0.04). Of the 56 patients of OPC and carbamate poisoning, 13 patients (23.2%) had respiratory arrest and required respiratory support. Time lapse had a significant role on the mortality in cases of acute poisoning (χ^2^ = 10.9, *P* = 0.01).

**Conclusion:**

Poisoning is more common in young males. The overall mortality is substantially high, mainly contributed by self-poisoning with insecticides and corrosives. Early care in a tertiary care center may help to reduce mortality in India.

## Introduction

Acute poisoning is an important medical emergency. The nature of poison used varies in different parts of the world and may vary even in different parts of the same country depending on the socioeconomic factors and cultural diversity. Management of these critically ill patients will greatly improve if the common causes of poisoning are properly defined.[1]

Pesticide self-poisoning accounts for about one-third of the world's suicides. Official data from India probably underestimate the incidence of suicides. The proportion of all suicides using pesticides varies from 4% in the European region to over 50% in the Western Pacific region, but this proportion is not concordant with the volume of pesticides sold in each region; it is the pattern of pesticide use and the toxicity of the products, not the quantity used, that influences the likelihood that they will be used in acts of fatal self-harm.[2]

With the progress in the industrial and agricultural field and advances in medical sciences a vast number of insecticides have become available, which on exposure may produce severe toxicity. Information available in our country is limited, with regard to acute poisoning in adults, including hospitalized patients.[3–7] In general, accidental poisoning is more common in children, whereas suicidal poisoning is more common in young adults.[3] A study from Vellore has shown an increasing trend of self-poisoning, especially among young adults.[7] It is important to know the nature and severity of poisoning in order to take appropriate preventive measures. Studies of this nature will be a useful tool in planning and management of critically ill acute poisoning cases. In this context the present study was carried out with the objective to investigate the pattern of acute poisoning cases in a tertiary care hospital in Karnataka.

## Materials and Methods

This retrospective hospital record-based study was conducted in a tertiary care hospital attached to a medical institution in Karnataka. The study included 136 cases of various acute poisoning due to drugs and chemicals in people above the age of 12 years in the year 2002. Cases of snake bite were also included in the study. But cases with food poisoning and allergic reactions to drugs were not included in the study. Data regarding age, sex, time elapsed after intake; circumstances of poisoning, name of poisonous substance, chemical type, duration of hospitalization, severity and outcome were collected in the prestructured proforma. Circumstantial evidences such as empty bottles and tablets were also collected from the patients. Data was collected for general physical examination and systemic examination of the patient. The data collected was entered in the computer database. Analysis was done by using proportion and chi-square test.

## Results

A total of 136 patients of various poisoning cases were studied. Incidence was more common among males (75.4%) compared to females (24.3) with a ratio of 3:1. Most cases of acute poisoning presented in the age group between 20 and 29 years (31.2%) followed by 12- to 19-year age group (30.2%). By occupation, 44.8% of the cases were manual laborers (61) followed by housewives (13.2%, 18), students (12.5%, 17), farmers and unemployed (10.2%, 14) and businessmen (8.8%, 12).

A majority of the poisoning cases (36.0%) were due to organophosphorus compound (OPC) followed by snake bite (16.2%), drugs (11.0%), rat poison (7.3%) and others. Drugs used were phenobarbitone, diazepam, alprazolam, cough syrups and mixture of tablets/capsules. All patients with rat poison had jaundice secondary to hepatotoxicity, whereas only one patient with drug poisoning had hepatotoxicity. Corrosives were acids and kerosene. Total mortality was found to be 15.4% (21). Mortality rate was 62.5% among patients with corrosive poisoning followed by a mortality of 26.5% in OPC. Patients who died due to OPC (13 cases) had respiratory arrest (9 cases), pneumonia and septicaemia (3 cases) and sudden cardiac arrest (1 case). Two patients had aspiration pneumonia and one had nosocomial pneumonia. Mortality in snake bite poisoning (2 cases) was because of respiratory paralysis (1 case) and severe hemorrhage (1 case). There was no mortality in organocarbamate and organochlorine compounds. Mortality rate due to corrosives was significantly high compared with OPC poisoning (χ^2^ = 4.12, *P* = 0.04) [Table 1].

**Table 1:** Types of poisoning and mortality

Type of poisoning	Number of patients (%)	Mortality (%)
Organophosphorus	49 (36.0)	13 (61.9)
Snake bite	22 (16.2)	2 (9.5)
Drugs	15 (11.0)	0 (0)
Rat poison	10 (7.4)	0 (0)
Corrosives	8 (5.9)	5 (23.8)
Organocarbamates	7 (5.1)	0 (0)
Organochlorine	5 (3.7)	0 (0)
Miscellaneous (plant and unknown)	20 (14.7)	1 (4.8)
Total	136	21

Maximum patients (7) expired when there was a delay in admission to hospital by more than 8 hours after ingestion, followed by a time period of 5-8 hours (6). Patients admitted within 2 hours of ingestion had the least mortality (2). Time lapse had a significant role in the mortality in cases of acute poisoning (χ^2^ = 10.9, *P* = 0.01) [Table 2]. The relationship between time lapse of more than 5 hours and mortality pattern among males and females are comparable in each type of poisoning [Table 3]. First aid was not found to be significant in minimizing the mortality of patients (*P* = 0.768). A total of 13 (13.3%) and 8 (21%) patients expired out of total 98 patients who received first aid and 38 patients who did not receive first aid, respectively. When we subdivided the cohort according to the type of poison, again the role of first aid did not have any significant bearing on the outcome.

**Table 2:** Time elapsed since exposure to hospital arrival and mortality

Time lapse (hours)	Total cases	Expired cases (%)
< 2	30	2 (6.7)
2-4	38	5 (13.2)
5-8	28	6 (21.4)
> 8	22	7 (31.8)
Unknown	18	1 (5.6)
Total	136	21 (15.4)

χ^2^ = 10.9, *P* = 0.01

**Table 3:** Relationship between time lapse and mortality of each type of poisoning among males and females (N = 50)

Type of poisoning	Time lapse (5 hours and more)		Mortality	
		
	Males (N)	Females (N)	Male (%)	Female (%)
Organophosphorus	15	5	6 (40.0)	2 (40.0)
Snake bite	3	1	1 (33.3)	0
Drugs	4	1	0	0
Rat poison	3	1	0	0
Corrosives	3	2	2 (66.7)	1 (50)
Organocarbamates	2	1	0	0
Organochlorine	1	1	0	0
Miscellaneous (Plant and unknown)	4	3	1 (25.0)	0
Total	35	15	10 (28.6)	3 (20.0)

It was found that 77.9% (106) of cases were of intentional poisoning for suicidal attempt and 22.1% (30) of cases had accidental poisoning. A majority (73%) of accidental poisoning were due to snake bite. Of a total of 21 patients (15.4%) who expired, 2 (9.5%) were secondary to accidental poisoning and the remaining 19 (90.5%) were secondary to intentional poisoning. Median hospital stay was 4 days. Only 13 patients (9.6%) stayed in the hospital for more than 15 days.

Eighty-one patients (60%) underwent psychiatric workup and were given psychiatric counseling and drug therapy. Reactive depression was seen in 48 (35%) patients secondary to failure in academic, social and financial areas and crisis in interpersonal adjustment. Other contributory factors were chronic alcoholism (21, 16%), financial stress (7, 6%) and manic depressive psychosis (5, 4%). Patients without psychiatric assessment were those who expired, were discharged at request or against medical advice.

## Discussion

In the present study, pesticides followed by snake bite were the two most common types of poisoning. A study conducted in Pondicherry revealed a rapidly increasing trend in the incidence of OPC poisoning over a 3-year period.[8] Other studies also showed that OPC are the most commonly used poisoning substances.[4,7] In contrast, some other studies showed that majority of poisoning admissions were due to pharmaceutical agents.[3,9] A study conducted at the All India Institute for Medical Sciences, New Delhi, showed that drugs (18%) and insecticides (12.80%) are the most common agents out of a total of 726 poisoning cases. Out of this insecticide group, carbamate (47) formed the largest group followed by OPC (43) and organochlorine compounds.[3] This difference in the type of poisoning seen within the country may be due to the difference in the pattern of use and availability of pesticides.

In this study, majority of the poisoning cases presented between 12- and 29-year age group (84, 61.7%). Similar findings were observed in other studies.[3–5] Males dominated the present study with male to female ratio of 3:1. However, some other studies have shown that males are marginally higher compared to females[5,6] and marginally more among females in others.[10,11] This high proportion of poisoning among males might be due to change in the lifestyle and cultural patterns in this area and other studies. In our study, the overall mortality was found to be 15.4%. Similar data were also obtained by a study which reported an overall mortality rate of 17.3%.[1] Other studies showed it as 3% - 4%.[7,9] Mortality in the present study is probably higher because of a higher number of pesticide and corrosives poisoning cases and higher rate of complicated cases.

It was seen from our study on psychiatric assessment, that majority of the suicidal cases were associated with reactive depression. High degree of stress in academic, financial and social sectors as well as inability to achieve the targets on professional, educational and socioeconomic fronts leading to limited alternatives were the contributory factors in taking suicidal actions. Similar factors were observed by others.[8,12,13] Majority of the patients (78%) consumed the poison with suicidal intent as compared with 22% of the patients exposed accidentally. A study conducted in Kathmandu (16-65 years age group) reported that 97% of the poisoning cases admitted in a hospital were due to suicidal attempt.[11] However, this study did not include snake bite cases unlike in our study. In contrast, another study done at New Delhi highlighted that nearly half (47%) of poisoning cases were accidental (1-70 age group).[6] But this study had included pediatric cases also unlike in our study in which we included only adolescents and adults.

It was found in our study that time lapse has a significant bearing on the total outcome. This is in comparison[13,14] and contrast[1] to other studies. In contrast, there was no significant difference in mortality of the patients with and without first aid. This is mainly because the patients who received first aid at small peripheral hospitals, were referred by a doctor after development of complications, which could not be managed at the peripheral centers. The first aid given had considerable variations in the administration of gastrointestinal lavage, dosage schedule of various antidotes such as atropine, PAM and anti-snake venom, etc, at the referral hospital. People who did not receive first aid were probably brought to the hospital directly as they may have been geographically closer to this hospital, and hence administered appropriate treatment earlier compared to others. As it was a retrospective study, it was difficult to draw firm conclusions regarding the role of first aid in acute poisoning. We feel that a prospective multicentric study with uniform criteria regarding first aid will give a final answer regarding the role of first aid at the level of primary health care.

The retrospective record-based nature and relatively small sample size are the limitations of our study. Some of the information such as time lapse for some patients, miscellaneous poisoning and types of snakes were not there in the records for analysis. Overall, the current study has managed to contribute substantial additional information regarding the epidemiology and outcome of poisoning in a tertiary care hospital at a district level. Poisoning is more common in young males. The overall mortality is substantially high, mainly contributed by self-poisoning with insecticides and corrosives. Timely transport and intervention of all critically ill poisoning cases is required to prevent the high mortality among victims. Educational and legislative interventions may be required to make the changes. There is a need to investigate further the high mortality rates associated with poisoning.
